# Whole-genome metabolic model of *Trichoderma reesei* built by comparative reconstruction

**DOI:** 10.1186/s13068-016-0665-0

**Published:** 2016-11-21

**Authors:** Sandra Castillo, Dorothee Barth, Mikko Arvas, Tiina M. Pakula, Esa Pitkänen, Peter Blomberg, Tuulikki Seppanen-Laakso, Heli Nygren, Dhinakaran Sivasiddarthan, Merja Penttilä, Merja Oja

**Affiliations:** 1 VTT Technical Research Centre of Finland, Tietotie 2, P.O. Box FI-1000, 02044 Espoo, Finland; 2 Department of Computer Science, University of Helsinki, P.O. 68 (Gustaf Hällströmin katu 2b), 00014 Helsinki, Finland

## Abstract

**Background:**

*Trichoderma reesei* is one of the main sources of biomass-hydrolyzing enzymes for the biotechnology industry. There is a need for improving its enzyme production efficiency. The use of metabolic modeling for the simulation and prediction of this organism’s metabolism is potentially a valuable tool for improving its capabilities. An accurate metabolic model is needed to perform metabolic modeling analysis.

**Results:**

A whole-genome metabolic model of *T. reesei* has been reconstructed together with metabolic models of 55 related species using the metabolic model reconstruction algorithm CoReCo. The previously published CoReCo method has been improved to obtain better quality models. The main improvements are the creation of a unified database of reactions and compounds and the use of reaction directions as constraints in the gap-filling step of the algorithm. In addition, the biomass composition of *T. reesei* has been measured experimentally to build and include a specific biomass equation in the model.

**Conclusions:**

The improvements presented in this work on the CoReCo pipeline for metabolic model reconstruction resulted in higher-quality metabolic models compared with previous versions. A metabolic model of *T. reesei* has been created and is publicly available in the BIOMODELS database. The model contains a biomass equation, reaction boundaries and uptake/export reactions which make it ready for simulation. To validate the model, we dem1onstrate that the model is able to predict biomass production accurately and no stoichiometrically infeasible yields are detected. The new *T. reesei* model is ready to be used for simulations of protein production processes.

**Electronic supplementary material:**

The online version of this article (doi:10.1186/s13068-016-0665-0) contains supplementary material, which is available to authorized users.

## Background

*Trichoderma reesei* is a filamentous fungus widely used for commercial scale production of biomass-degrading enzymes. Cellulose is the most abundant organic compound in the biosphere and is used as a raw material by many industries such as paper, food, textile and for biofuel production. *Trichoderma reesei* is the main industrial source for cellulases and hemicellulases, enzymes that hydrolyze the cellulose component of lignocellulosic materials. There is a need for reducing the cost and maximizing the yield of these cellulose-degrading enzymes.

Whole-genome stoichiometric metabolic models aim to fully explain the metabolism of an organism. The model is a collection of interconnected metabolic reactions that represent the biochemical possibilities of the organism. Whole-genome metabolic reaction networks have been reconstructed for many species, such as human (Recon 2) [1], yeast *Saccharomyces cerevisiae* [2], *Pichia stipitis* [3], *Pichia pastoris* [3], model plant *Arabidopsis thaliana* [4], bacteria *Escherichia coli* [5], *Bacillus subtilis* [6], fungus *Aspergillus niger* [7], *Aspergillus oryzae* [8], *Aspergillus nidulans* [9] and cyanobacteria *Synechocystis* [10]. To date, the BIOMODELS database [11] contains 1483 published models, some of which are whole-genome stoichiometric metabolic models. Additionally, the Path2models branch of the BIOMODELS database hosts an automatic reconstruction of 2641 whole-genome models based on the pathway information already included in KEGG [12] or MetaCyc [13] for the particular organism in question.

Metabolic modeling can aid as a tool in the development of microbial strains capable of high efficiency production of chemicals or proteins. Metabolic models are used to simulate the performance of the production strain in different scenarios (genetic modifications, cultivation set-ups) [14, 15]. High-quality metabolic models are required for successful metabolic simulations and predictions. So far, metabolic modeling for protein production scenarios has been rare, probably because the lack of good quality metabolic models for typical protein production hosts. Metabolic modeling of super oxide dismutase production in *Komagatella phaffii (P. pastoris)* has been done [16]. Metabolic modeling of protein produciton in *T. reesei* has been reported the first time in our accompanying paper [17], where the metabolic model from this paper has been used.

The reconstruction of metabolic models can be a tedious process including as many as 96 steps [18]. The main steps include the annotation of enzymes encoded by the organism’s genes, and the subsequent assembly of the metabolic reactions that are supported by these enzymes into a network. Recently, several automatic reconstruction tools have been proposed. Model SEED [19] is a web based pipeline for creating and analyzing metabolic models using techniques that automate the process. RAVEN [20] is a program suite for semi-automated reconstruction of a metabolic model based on a reference whole-genome metabolic model (or KEGG). RAVEN includes tools for gap-filling, quality control, compartmentalization and visualization of the models to speed up the manual curation work required on top of the automatic reconstruction step.

**Fig. 1:**
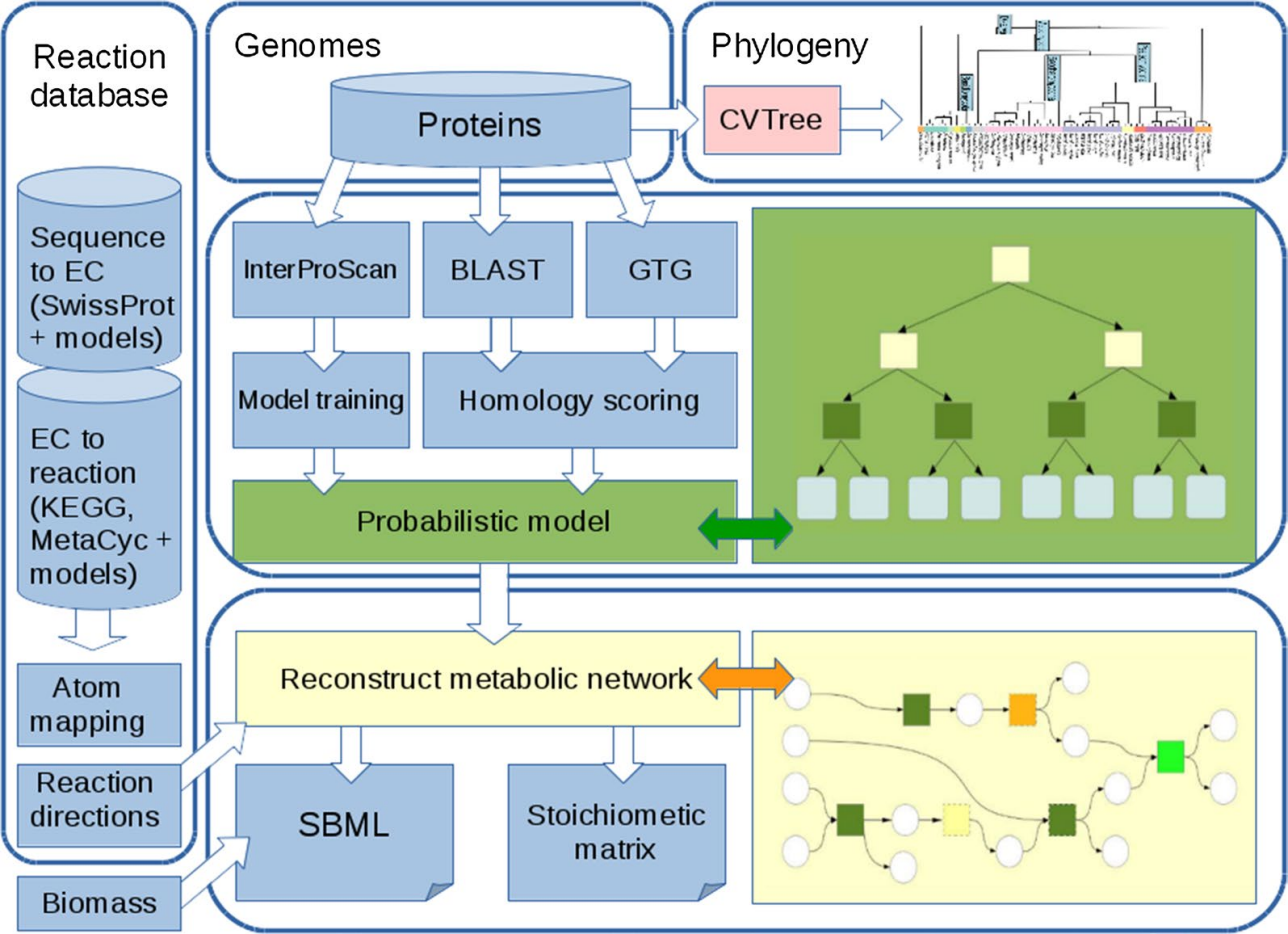
CoReCo metabolic model reconstruction pipeline. The algorithm has three main inputs: (1) genomes of the organisms (set of proteins), (2) phylogeny of the organisms, and (3) reaction database containing lists of metabolic reactions, reaction directionalities, and sequences of the enzymes catalyzing the reactions

The CoReCo algorithm [21] automatically reconstructs gapless metabolic models for several related species at once. The CoReCo metabolic model reconstruction pipeline has two parts (see Fig. 1). In the first part of the pipeline, the enzyme content of the input organisms is scored. The score for each enzyme is computed based on a probabilistic model that combines homology-based scoring from BLAST [22] and GTG [23] to InterPro annotations [24]. Including the phylogeny of the input organisms improves the prediction of enzymes in these organisms. In the interface between the two parts, a score for each reaction is computed as the maximum of the scores of the enzymes catalyzing the reaction. In the second part of the algorithm, a gapless metabolic model is created by adding high scoring reactions to the model, ensuring at each step the connectivity of the network. Low-scoring reactions may be added to avoid gaps. Enforcing gaplessness in the network reconstruction process and taking into account atom mappings [25] results in good quality models. However, the performance of previous CoReCo models had two main caveats: a number of cofactors or their precursors like biotin, pantothenate and choline could not be synthesized by the models and when testing for production of individual molecules slightly higher than stoichiometrically possible yields were often detected. Such problems were also common in early manually reconstructed metabolic models.

In this work, we present several improvements to the CoReCo metabolic model reconstruction method. CoReCo creates metabolic models by combining reactions given to it as inputs. So far, the KEGG database has been used as the source of reactions. Unfortunately, it has proven to be inadequate in several ways (1) several reactions are missing, (2) reactions are not balanced or contain undefined number of atoms (generic ‘R') or (3) are electron imbalanced. Electron imbalance negatively affects the calculation of reaction directions and flux balances. Missing reactions cause unnecessary gaps or long detour pathways. To resolve these issues, a new comprehensive database of metabolic reactions was created by combining information from public databases and whole-genome metabolic models. Furthermore, reaction directions were included as additional constraints for the gapless network reconstruction step of the algorithm. The scoring of enzymes was updated from previously having been the mean of the two evidence sources (GTG and BLAST) to being the maximum of the two evidence sources.

The whole-genome metabolic models can be used to simulate bioprocesses, for example, in a protein production set up. Central to metabolic modeling of bioprocesses, is the simulation of cell growth. To do this successfully, the cellular biomass composition needs to be described accurately in the metabolic model [26]. Cell growth is coupled to the metabolic network via a biomass equation that lists the components required for growth: amino acids, cell wall components, RNA, DNA, etc. Biomass composition has previously been measured and used to create biomass equations for genome-scale metabolic models of *S. cerevisiae* [27], *Scheffersomyces stipitis* [28] and *A. thaliana* [4]. The biomass equations in whole-genome metabolic models are similar to each other, but there are species specific differences.

The updated CoReCo metabolic model reconstruction pipeline was used to create a whole-genome metabolic model for the industrially important fungus *T. reesei*. CoReCo draws power from a phylogeny of input organisms, thus the *T. reesei* model has been reconstructed as part of a set of 56 fungi, allowing comparison of the fungal metabolic models. To complement the *T. reesei* metabolic model, we measured the biomass composition of *T. reesei*.

## Results

### Database of balanced reactions

Metabolic reactions from several public reaction databases and numerous relevant genome-scale metabolic models were gathered to create a high-quality database of balanced metabolic reactions. KEGG [12] and MetaCyc [13] list metabolic reactions in pathways, possibly leading to gaps between disconnected pathways. The whole-genome metabolic models of biotechnologically relevant fungi were included to provide a gapless set of metabolic reactions.

To combine the reactions from several different sources, first a unified list of all metabolites included in these reactions was created. Compounds were collected from YMDB [29], HMDB [30], ChEBI [31], KEGG Compound [12], and Rhea databases [32]. Metabolites from whole-genome metabolic models were mapped via database crosslinks, or via metabolite names when name-based matching allowed unique mapping. However, from each source, various metabolites remained unmapped, thus resulting in reactions not having all reactants mapped; see column “% of reactions fully resolved” in Table 1.

**Table 1:** Shows the origin of the reactions added to the reaction database

Species	Source	Reactions	% of matched reactions	% of balanced reactions	Selected as representative	Representatives that balance
*S. cerevisiae*	ymn6.06	1888	96	90	1020	923
*A. niger*	iMA871	1399	57	95	266	262
*A. nidulans*	iHD666	1303	85	69	88	72
*A. oryzae*	iWV1314	2360	93	74	265	235
*K. (Pichia) pastoris*	iLC915	1423	88	91	237	207
*P. stipitis*	iSS884	1332	88	92	6	5
*P. chrysogenum*	iAL1006	1636	83	95	75	67
KEGG reaction	KEGG	9236	93	90	6618	6376
MetaCyc	MetaCyc	11,181	62	90	3145	3054
Total		31,758	77	88	11,720	11,201

A fully resolved reaction is a reaction having all reactants identified by metabolites in the updated database. All representative reactions balance elements other than hydrogen. Reactions that also balance electrons are denoted “reactions that balance"

For the purpose of reconstructing models for fungi, genome-scale metabolic models of *S. cerevisiae* (community model v. 6.06) [2], *A. niger* (iMA871) [7], *A. oryzae* (iWV1314) [8], *A. nidulans* (iHD666) [9], *K. (Pichia) pastoris* (iLC915), *P. stipitis* (iSS884) [3], and *Penicillium chrysogenum *(iAL1006) [20] were included. In addition to these, reactions from KEGG and MetaCyc databases were included. The corresponding reactions from the different sources were combined, leaving only one copy of each unique reaction. In an effort to keep track of the source of the reactions, a representative for each unique reaction was selected. The representative was selected preferably from the whole-genome models, then from KEGG, and finally from MetaCyc. Column “Selected as representative” in Table 1, lists the number of reactions selected from each source. We selected 1020 reactions from the *S. cerevisiae* model, and then additional reactions, not present in *S. cerevisiae* model, from the other whole-genome models. 6618 KEGG reactions, not present in the whole-genome models, were selected. And finally, 3145 MetaCyc reactions, not present in the other sources, were included. Note that even though only 1020 of the 1888 reactions from the yeast *S. cerevisiae* model were selected, a good coverage of the core metabolism is achieved. The *S. cerevisiae* model includes identical reactions in several compartments and which have been combined in our reaction database. Lipid metabolism is not well-represented in neither the public databases (KEGG and MetaCyc) nor the whole-genome models. For the CoReCo reaction database, reactions for lipid metabolism were collected from v7.0 of the yeast community model [33]. Lipid reactions from other sources were removed.

Surprisingly, a large fraction of the metabolic reactions from the various sources showed problems in atom balances or charges (see column “% of balanced reactions” in Table 1). To tackle the issue of charge imbalance, the number of hydrogen atoms was replaced with the total amount of electrons in the compound. For some reactions, atom and electron balances were achieved using a balancing procedure that changed the stoichiometric coefficients while allowing the addition of water to the reaction equations. Still, some of the selected reactions had to be rejected due to balancing problems (compare columns “Selected as representative” and “Representatives that balance” columns in Table 1). Correct atom balances are required for the atom-mapping procedure, where each carbon atom in the substrates is mapped to the corresponding atom in the products [34]. Atom-mapping constraints aid in the gapless network reconstruction step of the CoReCo algorithm. Electron balance is important for the computation of reaction directions via thermodynamics. For this work, reaction directions were extracted from the selected, manually curated, full-genome metabolic models. However, our set of atom- and electron-balanced reactions would be ready for thermodynamic calculations using good group-contribution methods, such as [35–38].

In the CoReCo metabolic model reconstruction pipeline, the sequence information is coupled to the metabolic reactions via E.C. numbers. Previously, the sequence to E.C. information was extracted from SwissProt under the assumption that the enzyme annotations in this manually curated section of the UniProt database are reliable. The sequence to E.C. information for whole-genome metabolic models was extracted from the reaction gene rules included in these models and added to the Swissprot-derived information.

### Improvements to the CoReCo algorithm

Two main updates have been made to the CoReCo algorithm. First, the scoring of enzymes has been refined. In the first step of the CoReCo algorithm, the proteins of the input organisms are compared to sequence information using BLAST and GTG. Using the homology search results, the probability of observing each enzyme is scored for each species. This score is based on the probabilistic model that takes into account both BLAST and GTG and it has been shown that the inclusion of GTG improves the reconstructions [21]. Previously, the score was computed as an average of these two sources of information. In some rare cases, the BLAST evidence was very good, but the GTG evidence low. Thus, resulting in a score so low that the reaction was rejected from the reconstructed model even though BLAST clearly found the enzyme to be present in the organism. Low GTG evidence typically arises when GTG found the right enzyme but not the phylogenetically closest one. For example, for “2.7.8.1 Choline/ethanolaminephosphotransferase 1” the best match by BLAST for fungal sequences in Uniprot is the *S. cerevisiae* protein P22140 EPT1, while GTG found the *Homo sapiens* protein Q9Y6K0 CEPT1 as the best match. Both EPT1 and CEPT1 carry out the same reaction, but CEPT1 (due to phylogenetic distance) has far lower sequence similarity, hence the GTG score is close zero. Consequently, 2.7.8.1 was excluded from the models. In the updated version of CoReCo, the score is computed as the maximum of the BLAST and the GTG scores.

The second update is in the network reconstruction step where reaction directions are now taken into account. In the second phase of the CoReCo algorithm, the metabolic network is reconstructed using an algorithm that creates gapless metabolic networks [21]. The reactions are added to the network in an iterative manner, starting from the highest-scoring reactions, and subsequently adding reactions until the remaining reactions have a score lower than a user-defined threshold $\alpha$. At each stage, the connectivity of the network is guaranteed by requiring the new reactions to be connected to the already established network as well as to a predefined list of source metabolites. To ensure connectedness, a limited number of low-scoring reactions may be added to fill potential gaps. In the updated version of the CoReCo algorithm, reaction directions are taken into account in the gap-filling process, thus ensuring that the network reconstruction is proceeding in a more sensible manner.

**Fig. 2:**
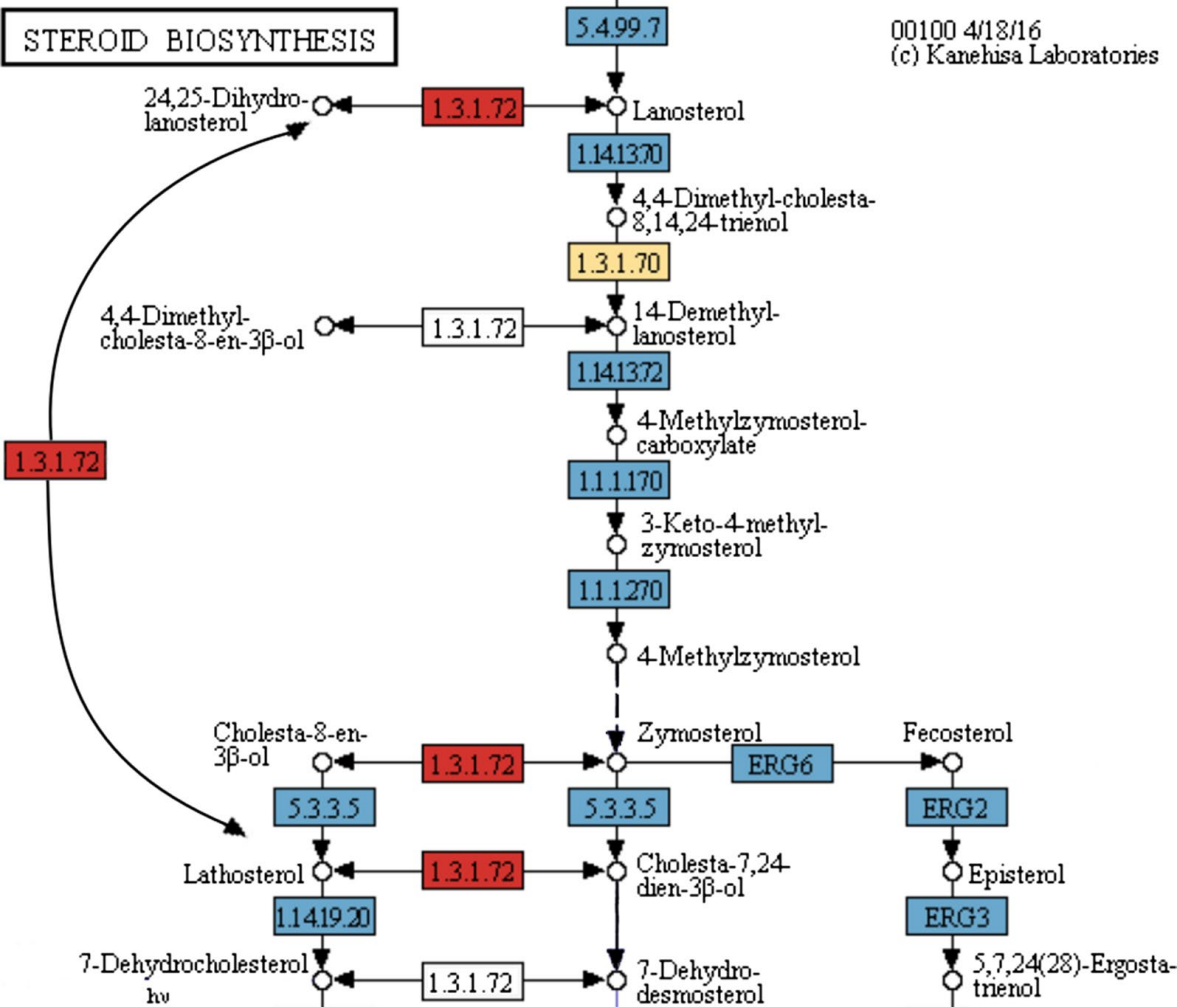
Example illustrating the benefit of adding reaction directions to the CoReCo gapless reconstruction step and improving the reaction database. The reactions that contain a *T. reesei* gene annotation in KEGG are colored *blue*. The reactions wrongly added in our previous metabolic model version for the production of zymosterol are colored *red*. The model presented in this work contains only the reactions colored *blue* and *yellow*

Zymosterol production exemplifies how the improvements made in the CoReCo pipeline and the reaction database result in a more accurate metabolic model. In KEGG’s zymosterol pathway as shown in Fig. 2, the reactions containing a *T. reesei* gene annotation are colored blue. Notice that the E.C.: 1.3.1.70, a $\delta$(14)-sterol reductase, is not found in *T. reesei* according to KEGG, however, CoReCo predicts that *T. reesei* contains E.C.: 1.3.1.70. The best evidence for CoReCo comes from the *T. reesei* gene tre81049 (JGI v2.0 genome annotation identifier), manually curated as C-14 sterol reductase that matches *N. crassa* erg-3 (P38670) in the bidirectional BLAST scoring scheme of CoReCo. *N. crassa* erg-3 has been manually curated to be a E.C.: 1.3.1.70 enzyme.

In the previous version of CoReCo, due to the fact that KEGG is missing reactions in the path going from 4-methylzymosterol to zymosterol, the CoReCo gap-filling step added low score reactions (marked red) to complete the path. The model connected lanosterol to lathosterol, and subsequently to zymosterol through the reactions with E.C. numbers 1.3.1.72 and 5.3.3.5. After reaction bounds were added to the completed model, it was unable to produce zymosterol since the reactions assigned to E.C. 5.3.3.5 are irreversible. However, after the reaction boundaries were included already in the gapless network assembly step and the additional use of the newly created reaction database, the reconstructed models in this specific case did not contain low-scoring reactions anymore and overall performed much better in similar situations.

The last step of the CoReCo pipeline is the writing of the models in SBML format. Exchange reactions, reaction directions and two biomass equations are included in each reconstructed metabolic model.

### Reconstruction of *T. reesei* metabolic model

A whole-genome metabolic model for *T. reesei* was built using the updated CoReCo algorithm and reaction database described above. CoReCo reconstructs models for several species at once by taking into account the phylogeny of the input species in the enzyme scoring step. Here, genomes of 56 fungi were used as input to CoReCo. This set includes the 49 fungi used in the first CoReCo publication [21]. For the purpose of reconstructing the *T. reesei* model, we added several *Trichoderma* genomes into the set of input organisms. A phylogeny of the input organisms was created using the CVtree algorithm [39]. For *T. reesei* metabolic model reconstruction, the CoReCo $\alpha$ parameter, i.e., the threshold for reaction scores was selected based on the biomass compound production in the simulations (see below). The CoReCo $\beta$ parameter, controlling how many reactions with a score lower than $\alpha$ may be added in the gap-filling step, was set to 2.

The reconstructed *T. reesei* model has 3926 reactions (including 148 exchange reactions and 261 orphan reactions) and 3348 metabolites. The orphan reactions are added to the network because their score exceeds the $\alpha$ parameter. However, the subsequent gap-filling process fails to connect them to the rest of the network. The automatically reconstructed model was able to create most of the biomass components. In the manual curation process, 16 reactions were added, rendering the model fully functional. Of the added reactions, 13 were related to lipid metabolism. These lipid metabolism reactions were not included automatically because they were not coupled to sequence information in the CoReCo input, and thus the algorithm was unable to include them. An additional reaction had to be included to allow the synthesis of l-threonine. This reaction’s score was just below the algorithm threshold. Finally, the P/O ratio was set to 1 by coupling ATP production to the reduction of oxygen in the electron transport chain adding two reactions. Correct reaction directions proved to be critical for a successful reconstruction. The reaction directions were originally harvested from the whole-genome models, but were iteratively updated during repeated rounds of reconstruction of models, simulating growth, and refining bounds. During this process, the boundaries of 112 reactions included in the *T. reesei* model were closed, and the direction of 24 reactions changed, respectively. The final models have been automatically reconstructed using the manually curated reaction directions.

### Construction of the biomass equation for *T. reesei*

A biomass equation is essential part of a metabolic model. It is necessary to have an accurate biomass equation, before the metabolic model can be used to simulate cellular metabolism and growth. To create the equation, the major components of biomass (total cellular protein, carbohydrates, DNA, RNA, esterified and free fatty acids and the major lipid classes) were measured in *T. reesei* grown on minimal medium containing cellobiose as a carbon source. The measurement data in this study was supplemented with data on the codon frequency in the transcriptome [17] to estimate the amino acid composition of proteins, and the amount of ash measured from chemostat cultures of *T. reesei* [40]. An overview of the biomass composition measurements is given in Table 2. The compound coefficients used in the biomass equation of the models can be seen in Table 3. The biomass equation was then coupled to the reconstructed metabolic model of *T. reesei*.

**Table 2:** The macromolecular composition of *T. reesei*

Biomass component			% (w/w)
Proteins			*45.100* ^a^
Ala			2.715^b^
Arg			3.944^b^
Asn			1.613^b^
Asp			2.677^b^
Cys			0.549^b^
Gln			2.063^b^
Glu			3.200^b^
Gly			1.649^b^
His			1.346^b^
Ile			2.186^b^
Leu			4.101^b^
Lys			2.595^b^
Met			1.202^b^
Phe			2.220^b^
Pro			2.363^b^
Ser			2.808^b^
Thr			2.354^b^
Trp			1.144^b^
Tyr			1.846^b^
Val			2.523^b^
Carbohydrates			*23.190* ^a^
Chitin			7.820^c^
Other carbohydrates			15.370^d^
RNA			*6.122* ^a^ ^b^
DNA			*0.912* ^a^
Lipids			*4.176* ^g^
Fatty acids-esters			
Myristic acid (C14:0) est			0.004^a^
Palmitic acid (C16:0) est			0.613^a^
Palmitoleic acid (C16:1n-7) est			0.011^a^
Stearic acid (C18:0) est			0.070^a^
Oleic acid (C18:1n-9) est			0.126^a^
Linoleic acid (C18:2n-6) est			1.425^a^
$\alpha$-linolenic acid (C18:3n-3) est			0.292^a^
Arachidic acid (C20:0) est			0.004^a^
Lignoceric acid (C24:0) est			0.005^a^
Fatty acids-free			
Palmitic acid (C16:0) FFA			0.060^a^
Stearic acid (C18:0) FFA			0.012^a^
Oleic acid (C18:1n-9) FFA			0.107^a^
Linoleic acid (C18:2n-6) FFA			0.274^a^
Ergosterol			0.278^a^
Triacylglycerol			1.792^e^
Phosphatidylethanolamine			0.551^e^
Phosphatidylcholine			1.102^e^
Ash			*5.100* ^f^

^a^Measured as described in "Methods" section

^b^ Amino acid ratios calculated based on codon ratios in RNAseq transcriptome data, with transcripts encoding secreted proteins removed

^c^ Estimated based on the chitin content (% w/w) of *A. oryzae* biomass [8], and corrected for different ash contents in *A. oryzae* and *T. reesei* preparates

^d^ Carbohydrates other than chitin

^e^ Calculated based on the measured ratio of TAG:PE:PC 52:16:32 and the measured amount of fatty acid esters

^f^ Measured from lactose-limited chemostat cultures of *T. reesei* Rut-C30 (*D* = 0.051/h) described in [40]

^g^ Sum of measured free fatty acids, ergosterol, triacylglycerol, phosphatidylethanolamine and phosphatidylcholine

### Biomass production by the reconstructed fungal metabolic models

The quality of the reconstructed metabolic models was assessed by simulations. To be able to use a metabolic model to simulate protein production, the model should be able to accurately predict cell growth and the load of protein production. The concept of growth is modeled via the biomass equation, whereas protein production load can be estimated as a combination of amino acids, ATP, ribonuclotides, and nucleotides required for the product protein, see for example [41]. Thus, to validate the quality of the models, we estimated their capability to produce the biomass components (including amino acids, ATP, RNA, and DNA). The goal of this analysis is to verify that the models contain all the necessary metabolic pathways.

**Fig. 3:**
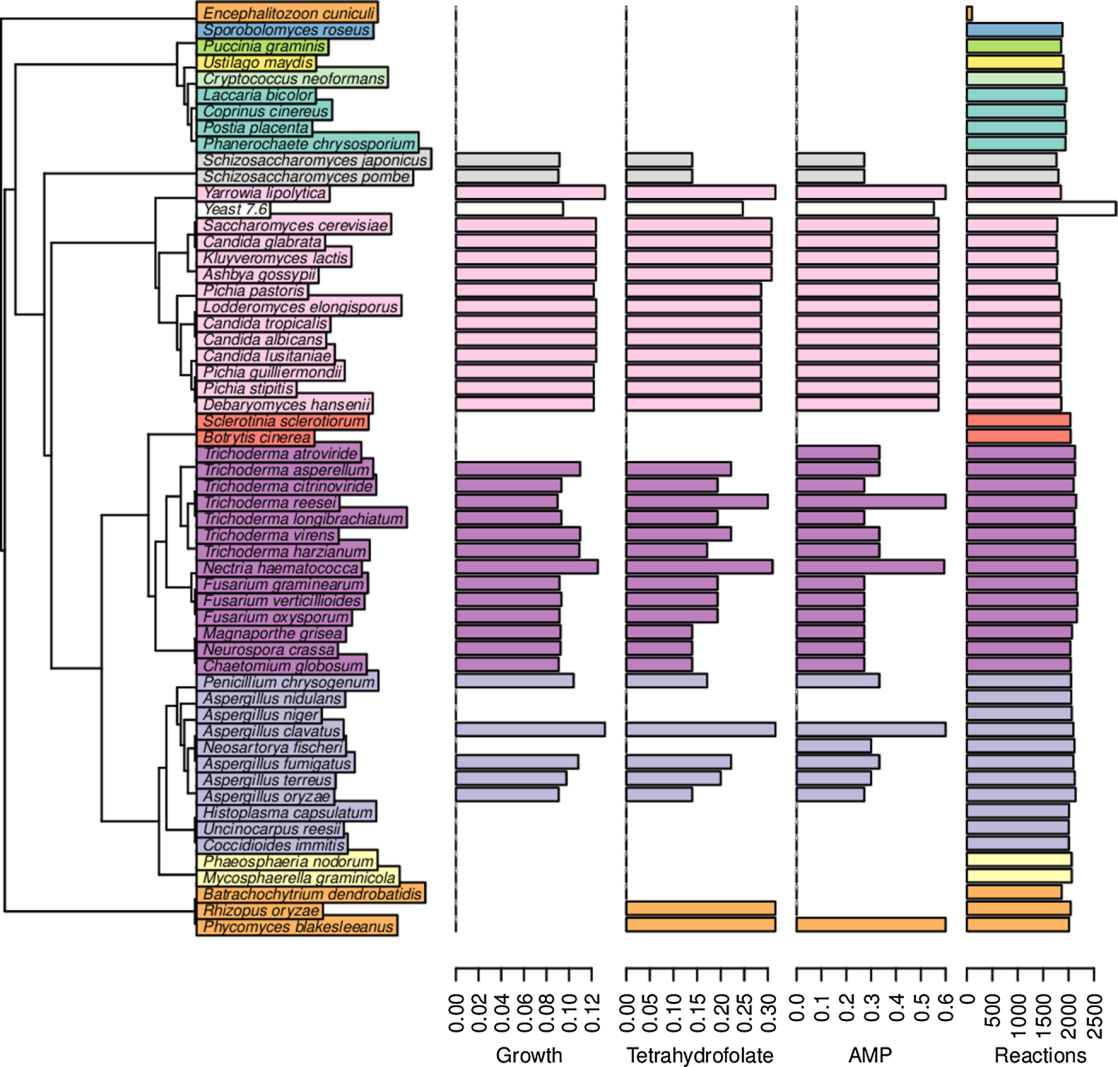
Overview of growth simulation results in the predicted fungal models. Shown on the *left* is the phylogenetic tree of the fungal species colored by taxons. Shown on the *right* are four sets of *barplots*: simulated growth rate (1/h), tetrahydrofolate production rate (mmol/g Cell Dry Weight (CDW) h), AMP production rate (mmol/g CDW h), respectively; and lastly, the number of reactions in the model (exchange reactions and orphan reactions are not included in this number). The rates are computed by FBA from 1 mmol/g CDW h of glucose uptake rate. Growth was simulated using the yeast biomass equation. Of the yeast biomass components, tetrahydrofolate and AMP are the two compounds most commonly unsuccessfully produced by the model, causing failure of simulating growth

First, the models’ ability to grow (Fig. 3), i.e., to produce all necessary biomass components in correct ratios, was tested with a simple biomass equation for *S. cerevisiae* from the model iMM904 [42]. The FBA was run such that the models were provided an input of a minimal media containing one unit of glucose and an unlimited amount of nitrogen, phosphate, water, oxygen, iron and sulfate. Models reconstructed with different reaction score inclusion thresholds ($\alpha$ parameter) were also compared. *T. reesei* model reconstructed with $\alpha$ *= 0.5* was able to produce the highest number of biomass components. With this $\alpha$ value, 33 species were able to grow in simulations. In contrast, with an $\alpha$ of 0.4 38 species were able to grow. The simulated growth rates, with $\alpha$ *= 0.5*, are shown in Fig. 3. In the simulations, *Saccharomycotina* species typically grow at a rate of 0.12, while *T. reesei* grows at a rate of 0.09.

**Fig. 4:**
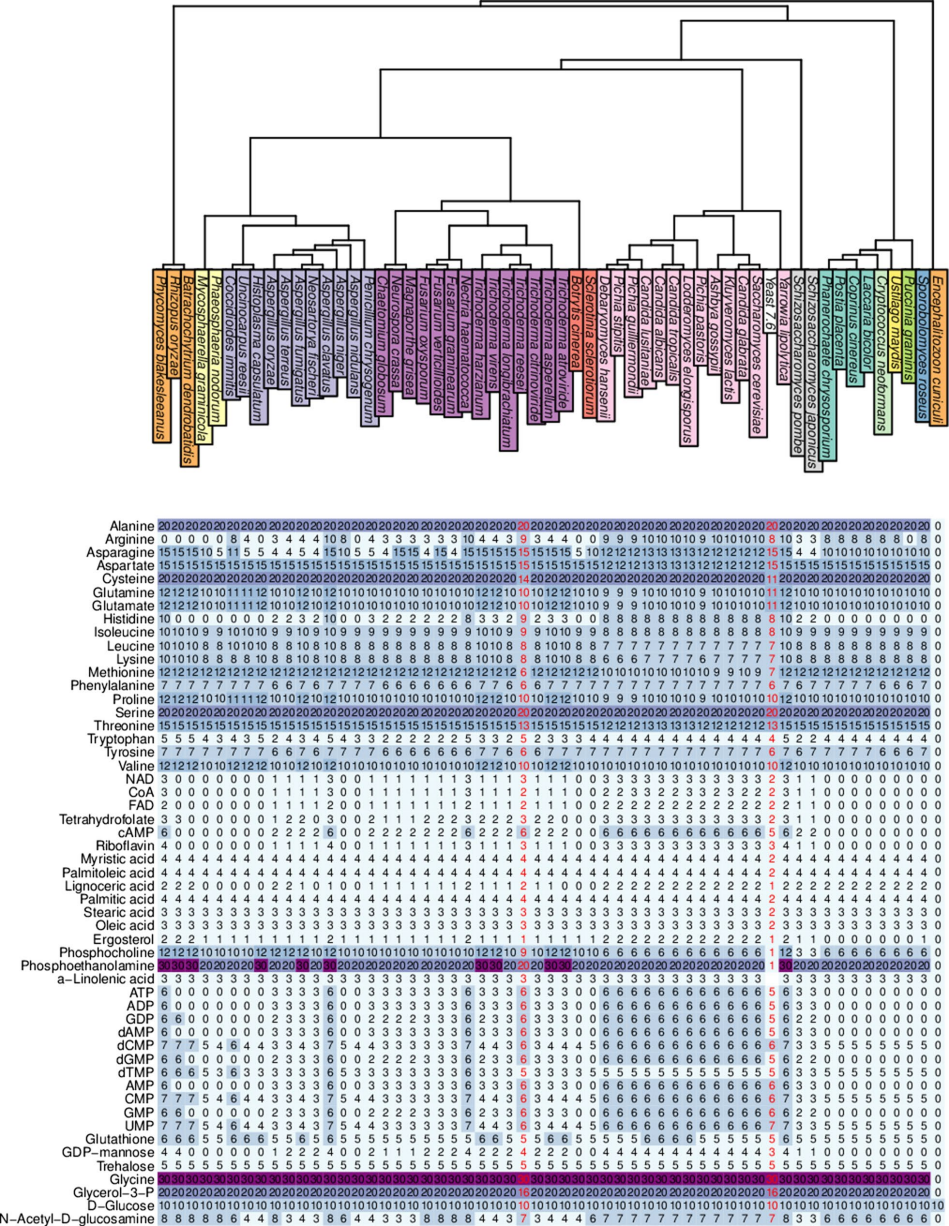
Production rates for components of the *T. reesei* biomass equation, simulated by FBA. As carbon sources, the models were given tenunits of glucose. Shown at the *top* is the phylogenetic tree of the fungal species colored by taxons. Shown *below* is a heatmap of production rates for each compound and species. For reference, the compound production rates have also been computed for the Yeast 7.6 model [43, 44]. Numbers have been rounded to integers

FBA was also carried out for simulating the production of each individual biomass compound included in the *T. reesei* biomass equation. For these simulations, the objective was set to maximize the production of a single biomass component, for example l-valine. As input, the models were given ten units of glucose and the same minimal media as described above. The simulation results are shown in Fig. 4 (see also Additional file 1) for a carbon normalized version of the same figure). In FBA simulations, the *T. reesei* model is able to produce all biomass components with stoichiometrically plausible yield, for example from ten units of glucose (C$_6$H$_{12}$O$_6$), the model creates 12 units of l-valines (C$_5$H$_{11}$NO$_2$).

Most of the other fungal metabolic models, created fully automatically using the same settings (reaction directions, addition of 13 lipid reactions, $\alpha$ *= 0.5*) as for *T. reesei*, are to a large extend able to create most of the biomass components as well (Fig. 4). Most importantly, the production rates for all tested compounds and in all species are stoichiometrically plausible, i.e., no extra carbon is created by physically unrealistic reactions. The biomass component production rates correlates with the phylogeny of the species. As a comparison, the identical FBA simulation setup was also applied to the Yeast 7.6 *S. cerevisiae* consensus model [43, 44] from http://yeast.sourceforge.net/. The compound yields are very similar in *T. reesei* and Yeast 7.6 with the notable exception of phosphoethanolamine and phosphocholine that are produced at far higher levels in the current CoReCo models. One compound, linoleic acid, currently included in the experimentally determined *T. reesei* biomass is not produced by Yeast 7.6.

### Curation of the automatically reconstructed metabolic model based on the literature

An extensive literature search about *T. reesei* was made to analyze the reliability of the reaction content in the metabolic model. A list of carbon sources was extracted from [45] (See Table 4). For each carbon source, an FBA was performed on the models maximizing for growth. The model achieved biomass production with each carbon sources tested, but five out of the 18 sources found in the literature were not included in the model.

Two cases could be identified, in which a specific enzymatic function was wrongly included in the model. *T. reesei* is known to lack the enzyme invertase needed to hydrolyze sucrose to form glucose and fructose [46, 47], as well as the enzyme glucose oxidase [45]. However, in the automatically created models, the reactions catalyzed by these enzymes were present. These wrongly assigned functions may arise from the influence of the phylogenetic tree on the final reaction scores, since many of the other *Trichoderma* species contain the enzymes that are missing in *T. reesei*. Both reactions were fixed manually in the model closing their bounds.

### Simulation of *T. reesei* metabolism

**Fig. 5:**
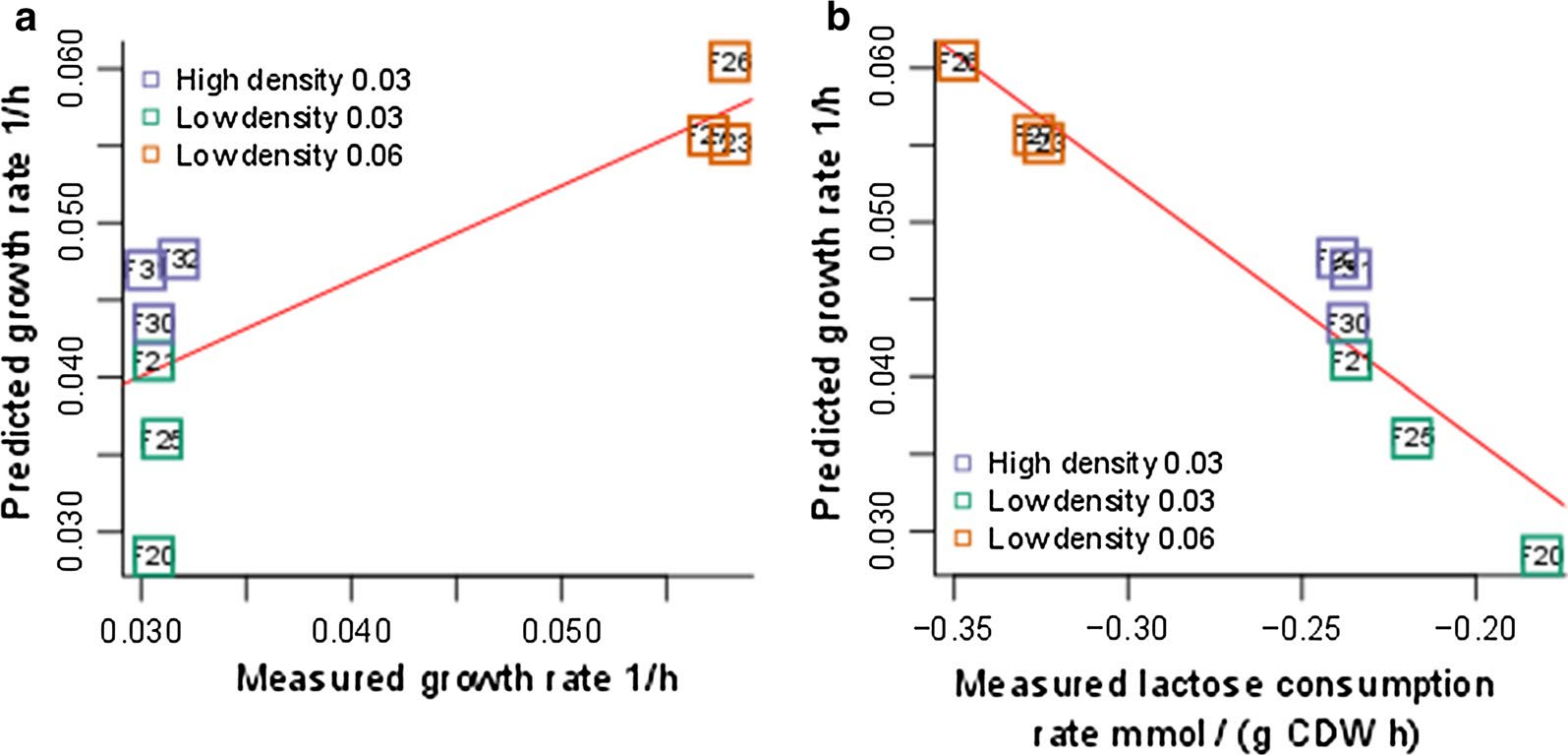
Growth rate simulations. On the *y*-axes, the predicted growth rate (h^−1^) and on *x*-axes **a** measured growth rate (h^−1^) and **b** measured carbon source, i.e., lactose consumption rate (mmol/(g CDW h)) are shown. The data points are *marked* with the fermentation identifiers (i.e., F32 is fermentation number 32) and surrounded by a *box*, *colored* to indicate the case corresponding to the fermentation. See further details from text

To assess the capability of the *T. reesei* model to predict the growth rate, chemostat cultivation data from a protein production experiment [48] was used for comparison. The model was constrained with the measured carbon source (lactose) consumption, CO$_{2}$ evolution and O$_{2}$ uptake rates. The cultivation data includes a set of nine chemostat cultures carried out under three different conditions: (1) high cellular density, growth rate of 0.03 h^−1^, (2) low cellular density, growth rate of 0.03 h^−1^, and (3) low cellular density, growth rate of 0.06 h^−1^. The high cellular density corresponds to a cellular dry weight (CDW) of 13 g/l and low cellular density to a CDW of 4 g/l. These conditions were selected to study the low growth rate protein phenotype of *T. reesei*, where the highest specific protein production rate is achieved at a relatively low growth rate of 0.03 h^−1^ and low cell density [49]. A Pearson correlation of 0.96 ($p<$ 0.0075) was found between measured and predicted growth rates (Fig. 5a). A slightly more significant Pearson correlation of −0.94 ($p<$ 0.0001) was found between predicted growth rate and measured lactose consumption rate (Fig. 5b). In case 1 (high cellular density, growth rate 0.03 h^−1^), the growth rate prediction is on average 0.015 h^−1^ higher than the measured rate. In case 2 (low cellular density, growth rate 0.03 h^−1^), the growth rate prediction is on average 0.004 h^−1^ smaller than the measured rate. In case 3 (low cellular density, growth rate 0.06 h^−1^), the growth rate prediction is on average 0.0005 h^−1^ larger than the measured rate. Furthermore, highest variation in the growth rate predictions is detected for case 2.

The assessment of this *T. reesei* model’s capability to predict protein production was carried out separately. The model was successfully used to predict production of native cellulases and of heterologous proteins in [17], where the experimental data used for the simulations is also thoroughly discussed.

## Discussion

The improvements to the CoReCo algorithm include the introduction of a better reaction database, update of the reaction scoring scheme, and inclusion of reaction directionalities into the reconstruction step. A new post-processing step adds exchange reactions, two optional biomass reactions, reaction bounds and the additional manually curated reactions (P/O ratio and required lipid reactions) to the models. With these additions the models are usable in simulations directly after being reconstructed.

### The quality of the reconstructed models

To successfully model cultivations, including protein production experiments, the model needs to have all the relevant biochemical pathways included. Amino acids and energy metabolism being especially relevant for protein production. It is also necessary to be able to accurately model cell growth during the bioprocess. To evaluate the quality of the models, and to assess the overall improvement of the database and the algorithmic updates to the simulation capabilities of CoReCo models, growth rate and individual biomass compound yields were simulated. Previously published CoReCo fungal models required trace amounts of eight different carbon containing compounds in the growth media of computational experiments to successfully simulate growth on the main carbon source, i.e., glucose. Even with these supplements only 17 out of 49 species could predict growth. With improvements presented in this work, 38 out of 56 species can grow and no other carbon containing compound, except glucose, is required in the medium. Furthermore, the biomass function of *T. reesei*, based on the measurements presented in this paper, contains ten more compounds than the previously used yeast biomass equation. The predicted growth rate of the new *T. reesei* model differs only by 0.004 h^−1^ from the simulated Yeast 7.6 growth rate. As measured in experiments, the maximal growth rate of *T. reesei* is lower than that of *S. cerevisiae*, but this is likely to result from other factors than stoichiometry.

The improvement presented in this work on the reaction database and the CoReCo algorithm has affected drastically the quality of the models (see a quantification of the effect of these improvements in Table 5). In previously published CoReCo fungal models [21], stoichiometrically unfeasible yields of biomass components were common. In average, 85% of the biomass compounds produced by the old models exhibited some level of stoichiometrically unrealistic yields, i.e., slightly more carbon was produced than was available from the carbon source. With the improvements presented in this work, for 3080 cases (55 compounds in 56 species), no stoichiometrically unfeasible yields were detected (see last row in Table 5). The number of reactions needed to fill the gaps in the networks (i.e., the number of low-scoring reactions) after adding the high-scored reactions was higher in the old models than in the new ones. In addition, the number of dead end metabolites and dead end reactions were higher in the old models compared with the new ones. We believe that the lack of reactions connecting some metabolites in the reaction database was forcing the algorithm to find alternative paths containing a major number of low scored reactions.

Furthermore, in comparison to Yeast 7.6 (Fig. 4) the biomass component yields of current CoReCo models appear very similar. The differences to Yeast 7.6 yields are likely to stem from problems in the reaction database. Hence, problematic cases like ethanolamine phosphate and choline phosphate production will give information for the starting point for the next round of reaction database improvement. However, real metabolic differences between the species could also be present.

When comparing the reconstructed fungal models with the Yeast 7.6 model as a benchmark, the *T. reesei* model appears to perform slightly better than our other reconstructed models. For example, the Yeast 7.6 model yields 11 units of cysteine from ten units of glucose, while *T. reesei* yields 14 units of cysteine and all other models 20 units, respectively. Similar issues can be seen for methionine, threonine and glycerol-3-phosphate. This is likely due to the fact that the reaction bounds assigned to the reaction database were evaluated based on the performance of the *T. reesei* model. Hence, although all the models are produced with the same automatic process, reaction database scuration is slightly biased towards producing a functional *T. reesei* model.

To assess the predictive capabilities of the CoReCo built *T. reesei* model, FBA was performed to simulate growth with experimentally determined rate data from protein production chemostat cultivations from [48] as input. The correlation between measured and predicted growth rates was good, but overall the model predicted a higher growth rate than what was measured (Fig. 5a). This is likely due to the fact that the energetically demanding protein production was not considered in this simulation. Protein production prediction has been made using the *T. reesei* model produced in this manuscript. The work made in [17] highlights with numerous examples how useful stoichiometric modeling can be for enzyme production. In particular, it was found both experimental and modeling based evidence for issues in sulfur assimilation.

Notably, in the condition where highest protein production occurs, the predictions show the highest variation. In this condition, the experimentally determined carbon source, i.e., lactose, consumption rate has lowest correlation to the measured growth rate. This discrepancy could stem from cellular regulatory choices of balancing the metabolism either towards growth or protein production. Further dissection of such discrepancies could lead to completely new insight on how to improve protein production.

## Conclusions

In this paper, we have presented algorithmic improvements to CoReCo, a tool for reconstructing whole-genome metabolic networks. The new models are more streamlined than previously: they have less reactions, and include reaction directionality constraints that render the solutions space for constrained based metabolic model applications smaller. The CoReCo software was updated such that the SBML-models received as output of the reconstruction pipeline are truly simulation ready containing a biomass equation, an objective function, and uptake and export reactions, respectively. We have demonstrated that the models can be used to simulate growth or metabolite production.

The CoReCo methodology was used to create a full-genome metabolic model for *T. reesei*. This model enables simulations of the metabolic aspects of protein production for this commonly used host organism in protein production applications. Simulations with the *T. reesei* model correspond well with experimental data. The applicability of the model for protein production has already been demonstrated in [17]. The model contains the reconstructed metabolic network, and a biomass equation that has been created based on the measurements of *T. reesei* biomass.

Current results indicate problem areas to which future improvement of the reaction database needs to be directed. With minor database improvement, CoReCo model reconstruction for all available genome sequences emerges as a tool to discover new stoichiometrically superior production organisms and pathways.

## Methods

### Updated database of metabolic reactions

Compounds were collected from YMDB, HMDB, ChEBI, KEGG Compound, and Rhea databases. Compounds from the different sources were mapped to each other based on database identifiers (KEGG_CID, ChEBI_CID, etc) and structure (e.g., InChI_string). Only metabolites for which the composition could be linked to a molecular data file (.mol file) were included in the updated database since a .mol file for each metabolite was required by the atom-mapping algorithm. Some sources contained only the names of the metabolites, i.e., the metabolites were not linked to any database identifier. If these metabolites could not be reliably mapped to compounds having a structure, the corresponding reactions from that particular source were left out of the updated database. For the purpose of the reconstruction algorithm, a unique identifier was selected to represent each metabolite. Information about the corresponding metabolite identifiers in the different sources was also kept.

A list of unique metabolic reactions was created by replacing the source-specific metabolite identifiers in reaction expressions with the unique metabolite identifiers mentioned above, and subsequently compared to one another. Reactions from different sources were deemed equal if they had the exact same set of participating metabolites. Protons were ignored during the comparison.

Out of the 11,720 unique reactions selected from the sources, 11,201 could be balanced to the level of electrons. The remaining reactions could only be element-balanced, but not electron-balanced. Despite the improvement made in the balancing algorithm, i.e., balancing the total number of electrons rather than balancing hydrogen atoms and charge, 519 metabolic reactions could not be fully balanced.

Reaction directions were harvested from the source whole-genome metabolic models. When conflicting reaction directions were encountered, the reactions were set to be bidirectional. KEGG- and MetaCyc-derived reactions were also set to be bidirectional, since these databases do not provide reliable information on the directionality of reactions. The reaction directions were iteratively updated until insensible flux distributions were no longer encountered in model simulations. After each update of reaction directions, the models were reconstructed anew using the updated reaction directions.

The reactions describing lipid metabolism in the updated reaction database were improved because various discrepancies with lipid reactions were noticed during tests with the CoReCo-reconstructed models. Metabolites were identified as lipids if they were part of the KEGG BRITE lipid database BRITE08002. Hence, all reactions containing these lipids were removed from the database, unless the source was yeast (version 7) or KEGG.

The yeast community model v. 7 was selected as the ’gold standard’, since the creators of the model [33] describe the lipid metabolism in detail, and provide scripts to update the lipid reactions from the yeast community model v. 6 to v. 7. The lipid reactions from other whole-genome metabolic models were removed from the updated reaction database. Only reactions having lipids with more than 20 carbon atoms needed to be updated.

All reactions entering the updated reaction database were named using identifiers from the source database. When possible, the KEGG reaction identifier was coupled to the reaction name from the source metabolic model; for example, r0226-YCM606-R00086 denotes the reaction r_0226 from the yeast community model (YCM606) corresponding to KEGG reaction R00086. KEGG and CHEBI identifiers were used as metabolite identifiers. In cases where the metabolite was not found from either source, a new identifier was composed. These CoReCo internal identifiers were called 'Cluster' followed by a number, for example, Cluster7157 ( *N*-carbamoylglycine) corresponds to entry 6988657 in PubChem and *N*-CARBAMOYLGLYCINE in MetaCyc.

### Updates to CoReCo algorithm

As described in the "Results" section, the improvements to the CoReCo algorithm include the introduction of a better reaction database, update of the reaction scoring scheme, and introduction of reaction directionalities in the reconstruction step. In addition to the above changes, a cluster version of the CoReCo algorithm was created to allow the user to run the pipeline in a parallel mode.

The SBML output file of the CoReCo model now includes exchange reactions (based on the yeast community model [42]), reaction bounds (harvested from the whole-genome models and also manually curated in the reconstruction) and two biomass equations. One biomass equation is based on the yeast community model, the other is the experimentally determined *T. reesei* biomass composition. This update renders CoReCo created models immediately suitable for simulations. The models were simulated in R using the sybil package [50] and in Matlab environment using the Cobra toolbox [51].

P/O ratio has been established by coupling the Ferrocytochrome-c:oxygen oxidoreductase (r0438-YCM606-R00081) and ATP phosphohydrolase (r0226-YCM606-R00086) reactions. The P/O ratio was set to 1.

### Reference sequence data

The CoReCo reactions scoring scheme is dependent on a reliable sequence to E.C. information. Here, SwissProt E.C. number annotations and gene rules from the input metabolic models were used as the reference. To create a database for protein sequence BLAST, protein sequences of the genes annotated as enzymes in the included metabolic models were combined to the protein sequences from SwissProt. A file containing the relationship between the protein sequence name and the E.C. number for all the sequences was created by combining the E.C. annotations of sequences in UniProt to the E.C. numbers annotated in the included models. In cases where the E.C. number assignment in SwissProt and in a whole-genome model were conflicting, both annotations were used. In cases where the E.C. assignment in Uniprot was in TrEMBL and not in SwissProt, only the E.C. from the source model was used. In some cases, the source whole-genome models included gene annotations for reactions without an E.C. In these cases, a pseudo E.C. number of the same format, i.e., 7.xx.xx.xx, was used instead to provide a coupling between the reaction and the gene information.

### Sequence data for *T. reesei* and other fungi

Instead of the actual genomes of the input organisms, CoReCo uses the full set of protein sequences from each organism. In each case, the protein sequence data for the species of interest were downloaded from JGI and the FASTA headers modified to have a clear identifier as the first word in the header. For 49 fungi, the FASTA sequences were already collected for the previous CoReCo run [21]. This set was complemented with the protein sets of *Trichoderma asperellum*, *Trichoderma atroviride*, *Trichoderma harzianum*, *Trichoderma longibrachiatum* and *Trichoderma virens*. Since the previous CoReCo run, an updated genome sequence for *Komagataella (Pichia) pastoris* has become available [52] and was used to replace the previous data for this organism.

The phylogenetic tree of the organisms was computed using the CVtree algorithm [39]. CVtree is an alignment free composition vector tree based method, and hence does not require selection of specific genes for phylogeny reconstruction. The only parameter required by the method is the length K of the oligopeptides, which was set to 7. The K parameter controls the resolution of the method and it is recommended by authors of the method to be set to 6 o 7 for fungi. We used the fully predicted proteomes of 67 fungi, and the choanoflagellate *Monosiga brevicollis* as an outgroup [53] and extracted a subtree for the 56 fungi from this tree.

### Biomass measurements in *T. reesei*

The biomass composition of *T. reesei* VTT D-00775 $\Delta$mus53 was analyzed to create the biomass equation in the model of *T. reesei* (The deletion of the gene mus53 is done to help further construction of modified strains by enhancing homologous recombination in the strain construction process.) The strain was cultivated as follows: 400 ml of culture medium 7.6 g/l (NH$_{4}$)$_{2}$SO$_{4}$, 15.0 g/l KH$_{2}$PO$_{4}$, 2.4 mM MgSO$_{4}\cdot$7H$_{2}$O, 4.1 mM CaCl$_{2}\cdot$H$_{2}$O, 3.7 mg/l CoCl$_{2}$, 5 mg/l FeSO$_{4}\cdot$7H$_{2}$O, 1.4 mg/l ZnSO$_{4}\cdot$7H$_{2}$O, 1.6 mg/l MnSO$_{4}\cdot$7H$_{2}$O, pH adjusted to 5.2 with KOH, and supplemented with 25 g/l cellobiose was inoculated with 8 $\cdot$ 10$^{7}$ spores, and cultivated in shake flasks on a rotary shaker (250 rpm) at 28 °C for 3 days. 100 ml of the preculture was transferred to Sartorius Q plus bioreactors containing 900 ml of the medium (4.4 g/l (NH$_{4}$)$_{2}$SO$_{4}$, 15.0 g/l KH$_{2}$PO$_{4}$, 2.64 mM MgSO$_{4}\cdot$7H$_{2}$O, 4.5 mM CaCl$_{2}\cdot$H$_{2}$O, 4.1 mg/l CoCl$_{2}$, 5.5 mg/l FeSO$_{4}\cdot$7H$_{2}$O, 1.54 mg/l ZnSO$_{4}\cdot$7H$_{2}$O, 1.76 mg/l MnSO$_{4}\cdot$7H$_{2}$O, 25 g/l cellobiose). Cultivation temperature was 28  °C. pH was adjusted to 4.8 ± 0.1 by addition of 15% KOH or 15% H$_{3}$PO$_{4}$. The dissolved oxygen saturation level in the cultures was >30%, agitation 500–1200 rpm with a tip speed of 1.1 to 2.7 m/s, and a total aeration flow of 0.6 l/min. The cultures off-gas was monitored on-line for CO$_{2}$ and O$_{2}$, and samples of the cultures were collected at 0, 16, 24, 40, 64, 88 and 112 h. The mycelial samples were separated from the culture supernatant by filtering through Whatmann GF/B filters and washing with an equal volume of water, frozen immediately in liquid nitrogen, and stored at −80 °C for further analysis. Culture supernatant samples were stored at −20 °C . For sugar analytics, 1.5 ml culture supernatant samples were acidified by the addition of 10 μl of 97% H$_{2}$SO$_{4}$ before storing. Analysis of carbon source consumption, growth and protein production in the cultures as well as transcriptome data is described in [17].

The samples collected at 40 h after inoculation of the bioreactors were analyzed for biomass composition. Three replicate cultures were analyzed.

Biomass dry weight of the cultures was measured by filtering and drying the mycelium at 105 °C to constant weight.

Analysis of DNA in the mycelium was analyzed essentially as described by [54]. 2.5 ml of ice cold 0.25 M HClO$_{4}$ was added to the samples of mycelium (10–30 mg of dry weight per assay). The samples were kept in an ice water bath for 30 min with occasional shaking, and then centrifuged. The supernatant was discarded, and the pellet was extracted by resuspending in 1 ml of 0.5 M HClO$_{4}$ by vortex, incubated at 70 °C for 15 min with occasional shaking, and finally the supernatant was separated by centrifugation. The extraction was repeated twice by resuspending the remaining pellet in 0.5 ml of 0.5 M HClO$_{4}$, vortexing, incubating at 70 °C for 15 min with occasional shaking. Supernatants from each extraction step were combined, the volume adjusted to 2.5 ml with 0.5 M HClO$_{4}$, and DNA measured using the diphenylamine method. 1–2 ml of sample was mixed with 2 ml of diphenylamine reagent containing acetaldehyde, incubated at 30 °C overnight, and the optical density measured at 600 nm. The result was compared with standards that were treated in the same way.

RNA content of fungal biomass was measured as described by [55]. Mycelium was washed three times by resuspending in 3 ml of cold 0.7M HClO$_{4}$ and centrifuging, after which the mycelium was resuspended in 3 ml of 0.3M KOH and incubated at 37 °C for 60 min with occasional shaking. After cooling to room temperature, the samples were neutralized by adding 1.0 ml 3M HClO$_{4}$, and centrifuged. The supernatant was collected, and the pellet was washed twice with 4 ml of cold 0.5M HC1O$_{4}$. The supernatants were combined, and the volume was adjusted to 15 ml with 0.5M HClO$_{4}$. Finally, the samples were cleared by centrifugation, and the absorbance A$_{260 nm}$ was measured.

Carbohydrate amount in the fungal biomass samples was measured using the phenol method, essentially as described by [54]. Mycelium samples were ground using a mortar and pestle under liquid nitrogen and lyophilized. 20–200  μg of lyophilized cells were resuspended in water. 1 ml of 5% phenol was added to the samples, the standards prepared from glucose, and to the reagent blanks (1 ml of water). 5 ml of concentrated sulphuric acid was added as a stream to the surface of all the tubes, while shaking the tubes simultaneously. The tubes were allowed to stand for 10 min at room temperature, shaken, and placed in a water bath at 25–30 °C for 10–20 min before measuring the absorbance at 488 nm.

The amount of cellular protein was measured as described by [54]. Mycelium samples were ground using a mortar and pestle under liquid nitrogen and lyophilized. Lyophilised mycelium, corresponding to 1–5 mg dry weight, was resuspended in 2 ml of water. 1 ml of 3M NaOH was added, and the samples were transferred to a boiling water bath for 5 min, and cooled in cold (+4 °C) water bath for 5 min. 1 ml 2.5% CuSO$_4$ was added, the samples were shaken thoroughly, let stand for 5 min, after which the samples were centrifuged, and the absorbance A$_{555 nm}$ was measured. A reagent blank containing 2 ml of distilled water instead of cell suspension, and a set of standard protein solutions were treated in the same way, including the heating step.

For extraction of lipids, freeze-dried mycelium (5 mg) was resuspended in 200  μl of 15 mM NaCl and spiked with internal standards [triheptadecanoate (50 μg) and heptadecanoic acid (25 μg)]. Extraction of lipids for fatty acid and lipid class analyses was performed with chloroform:methanol (2:1, 1000 μl). After vortexing and 30 min extraction time at room temperature, the samples were centrifuged at 10,000 rpm for 3 min. The extract (lower layer) was separated and evaporated into dryness under nitrogen flow, and dissolved into 1000 μl of petroleum ether (fatty acid samples) or 100 μl of dichloromethane (lipid class samples).

For GC-MS analysis of fatty acids, lipids were transesterified with sodium methoxide by adding 500 μ of 0.5 N NaOMe in MeOH and a couple of boiling stones and incubating the mixture at 45 °C for 5 min. The samples were acidified with 15% NaHSO$_4$ and the methyl esters as well as free fatty acids were extracted with petroleum ether. The separated petroleum ether layer was evaporated and dissolved into 100 μl of hexane. Fatty acid methyl esters were analyzed on an Agilent 7890A GC combined with an Agilent 5975C mass selective detector. The column was an Agilent FFAP silica capillary column (25 m $\times$ 0.2 mm $\times$ 0.3 μm). Helium was used as carrier gas with a split ratio of 15:1. The oven temperature programme was from 70 °C (2 min) to 235 °C at a rate of 10 °C/min, total run time was 30 min. The temperatures of the injector and MS source were 220 and 230 °C, respectively. The samples (2 μl) were injected by a Gerstel MPS injection system and the data were collected in EI mode (70 eV) at a mass range of *m/z* 40–600. After analyzing fatty acid methyl esters by GC-MS, the same samples were trimethylsilylated (TMS) to determine free fatty acid (FFA) and sterol contents. Samples were evaporated, dissolved into 30 μl dichloromethane (DCM) and silylated with 25 μl of MSTFA [*N*-methyl-*N*-(trimethylsilyl)trifluoroacetamide] at 80 °C for 20 min. Trimethylsilylated samples were analyzed by GC-MS on a Restek Rtx®-5MS column (15 m $\times$ 0.25 mm $\times$ 0.25 μm). The split ratio was 20:1 and the oven temperature programme from 70 °C (1 min) to 270 °C at a rate of 10 °C/min, the total run time was 30 min.

The lipid class analyses (HPLC-ELSD) were performed on a Waters Alliance HPLC combined with a Cunow DDL21 evaporative light scattering detector (ELSD). Separation of the lipid classes was carried out on a Waters Spherisorb®silica column (5 μm, 150 $\times$ 4.6 mm I.D.). The gradient system consisted of (A) MTBE (methyl tert-butyl ether)-tetrahydrofurane (99:1), (B) 2-propanol-DCM (dichloromethane; 4:1) and (C) 2-propanol-water (1:1) containing triethylamine and formic acid (50 μmol). The temperature of the detector was 40 °C and air flow 27 psi. The multigradient system started from 100% A, the proportion of A decreased to 32%, that of B increased to 52% and simultaneously that of the water containing C increased to 16%. Keeping the cycle running continuously enabled stable retention times. The injection volume was 30 μm.

### Construction of the biomass equation

The coefficients used in the biomass equation of the *T. reesei* model were calculated based on the measurement data on *T. reesei* biomass composition. The coefficients in the biomass equation indicate the concentration of the compound as mmol/g CDW. Additionally, a few coefficients were copied from the biomass equations of *S. cerevisiae* and *A. oryzae* as explained below.

The amount of cellular proteins [0.451 g/ (g CDW)] in *T. reesei* cultures was measured as described above. The molar ratio of amino acids in the proteins was calculated based on the codon abundance in the RNAseq data of transcriptome excluding 31 transcripts encoding secreted proteins that were identified based on 2D-gel analysis [48]. The RNAseq data was from *T. reesei* cultivated in similar conditions as the cultures for biomass measurements [17]. The molar ratios of the amino acids were converted to weight ratios using the formula weight of each amino acid subtracted by the formula weight of a water molecule released in peptide bond formation, and the weight ratios used for calculation of the amount of each amino acid in the cellular proteins, and subsequently in the fungal biomass (g/(g CDW)) (Table 2). The corresponding molar amino acid amounts were used as coefficients in the biomass equation (mmol/(g CDW)) (Table 3).

**Table 3:** The coefficients (mmol/g CDW) used in the biomass equation of the *T. reesei* model

Kegg Id	Compound name	Coefficient
C00133	Alanine	−0.382^a^
C00062	Arginine	−0.253^a^
C00152	Asparagine	−0.141^a^
C00049	Aspartate	−0.233^a^
C00097	Cysteine	−0.053^a^
C00064	Glutamine	−0.161^a^
C00025	Glutamate	−0.248^a^
C00037	Glycine	−0.289^a^
C00135	Histidine	−0.098^a^
C00407	Isoleucine	−0.193^a^
C00123	Leucine	−0.362^a^
C00047	Lysine	−0.202^a^
C00073	Methionine	−0.092^a^
C00079	Phenylalanine	−0.151^a^
C00148	Proline	−0.243^a^
C00065	Serine	−0.322^a^
C00188	Threonine	−0.233^a^
C00078	Tryptophan	−0.061^a^
C00082	Tyrosine	−0.113^a^
C00183	Valine	−0.254^a^
C06424	Myristic acid	−0.00015^b^
C08362	Palmitoleic acid	−0.00043^b^
C06427	$\alpha$−Linolenic acid	−0.01^b^
C00219	Arachidic acid	−0.00013^b^
C08320	Lignoceric acid	−0.00014^b^
C00249	Palmitic acid	−0.026^c^
C01530	Stearic acid	−0.003^c^
C00712	Oleic acid	−0.008^c^
C01595	Linoleic acid	−0.061^c^
C01694	Ergosterol	−0.007^d^
C00093	Glycerol−3−*P*	−0.04^e^
C00588	Phosphocholine	−0.014^e^
C00346	Phosphoethanolamine	−0.007^e^
C00031	d−Glucose	−0.385^f^
C00140	*N*-acetyl-d-glucosamine	−0.948^f^
C00360	Deoxyadenosine monophosphate	−0.007^g^
C00239	Deoxycytidine monophosphate	−0.008^g^
C00362	Deoxyguanosine monophosphate	−0.008^g^
C00364	Deoxythymidine monophosphate	−0.007^g^
C00020	Adenosine-monophosphate	−0.047^h^
C00055	Cytidine monophosphate	−0.045^h^
C00144	Guanosine monophosphate	−0.053^h^
C00105	Uridine monophosphate	−0.045^h^
C00001	Water	−59.276^i^
C00002	Adenosine triphosphate	−59.276^i^
C00008	Adenosine diphosphate	59.276^i^
C00009	Organic phosphorous	59.305^i^
C00059	Sulfate	−0.02^i^
C00255	Riboflavin	−0.001^i^
C00010	Coenzyme A	−0.000001^j^
C00003	Nicotinamide adenine dinucleotide (NAD)	−0.000001^j^
C00016	Flavin adenine dinucleotide (FAD)	−0.000001^j^
C00051	Glutathione	−0.000001^j^
C00101	tetrahydrofolate	−0.000001^j^
C00575	3′,5′-cyclic AMP	−0.000001^j^
C00096	GDP-mannose	−0.000001^j^
C01083	$\alpha$,$\alpha$-Trehalose	−0.000001^j^

The coefficients correspond to the measured or estimated molar amounts of the compound in the cells, as described in "Methods" section

^a^The amount of amino acids calculated based on the measured cellular protein and the ratio of amino acids in cellular proteins calculated based on the codon abundancy in the RNAseq data of transcriptome

^b^ Esterified fatty acid measured using GC-MS

^c^ Sum of measured esterified and free fatty acid measured using GC-MS

^d^ Measured using GC-MS

^e^ The amount estimated to be needed for synthesis of triacylglycerols, phophatidylethanolamines or phosphatidylcholines (1 mol of glycerol-3-*P*, phosphoethanolamine or phosphocholine per 1 mol of triacylglycerol, phosphatidylethanolamine or phosphatidylcholine, respectively)

^f^ The measured total carbohydrate was assumed to consist of polymers of d-glucose subunits (glucan) and polymers of *N*-acetyl-d-glucosamine (chitin). Chitin content of the cells was estimated based on the amount of chitin in *Aspergillus oryzae* [8], and the rest of the measured carbohydrate as glucan

^g^ The amount of deoxyribonucleotides in DNA calculated based on the cellular DNA amount and the GC content of the genome

^h^ The amount of ribonucleotides in RNA was estimated based on the measured total RNA amount the nucleotide ratio in genome region encoding ribosomal 18S-28S pre-rRNA

^i^ As in *S. cerevisiae* model iMM904 [42]

^j^ Trace amount of the compound was added

**Table 4:** Carbon sources used by *T. reesei* found in the literature

Carbon source	Growth rate (per unit of compound)
$\alpha$-methyl-d-mannoside	Not found in the model
$\beta$-methyl-d-glucoside	Not found in the model
Arbutin	0.171
Cellobiose	0.190
d-arabitol	0.080
d-Fructose	0.090
d-Galactose	0.095
d-Glucose	0.090
d-Mannitol	0.095
d-Mannose	0.090
d-Sorbitol	0.100
d-xylose	0.075
Esculin	Not found in the model
Glycerol	0.049
Glycerol-1-monoacetate	Not found in the model
l-arabinose	0.075
l-sorbose	0.095
*N*-acetyl-$\beta$-d-glucosamine	0.115
Salicin	0.185
Trehalose	0.190

The exchange reaction for each of these compounds has been opened to allow the model to have one unit of uptake. Growth has been maximized using FBA

**Table 5:** Comparison of the previously reconstructed models [21] with the new models produced by CoReCo in this article

CoReCo model	*T. reesei*		*S. cerevisiae*		Average of all models^a^	
	Pitkänen [21] (%)	This article (%)	Pitkänen [21] (%)	This article (%)	Pitkänen 21] (%)	This article (%)
Dead end reactions	58	44	61	44	59	44
Dead end metabolites	68	59	70	58	69	58
Reactions added during the gap-filling step	37	20	35	22	39	21
Biomass components produced	7	100	7	100	7	89
Biomass components produced*	98*	100	100*	100	91*	89
Biomass components produced with stoichiometrically unrealistic yield	98*	0	64*	0	85*	0

* When eight extra compounds were added as a source metabolites into the model simulations

^a^ The obligate intracellular parasite *Encephalitozoon cuniculi* has been excluded from the averages

Esterified and free fatty acids were measured using GC-MS as described above. The amount of triacylglycerols, phosphatidylethanolamines or phosphatidylcholines in the cells was calculated based on the ratio of triacylglycerols : phosphatidylethanolamines : phosphatidylcholines (0.52 : 0.16 : 0.32 (w/w/w)) determined using HPLC-ELSD and the measured amount of esterified fatty acids in the cells. The measured fatty acid residues were assumed to be distributed equally in the lipid classes, three fatty acid residues in triacylglycerols and two fatty acid residues in phosphatidylethanolamines and phosphatidylcholines. Total amount of the esterified fatty acids was 0.0995 mmol/(g CDW) and the average MW of the fatty acid esters in the pool was 274.2 g/mol, which was used for calculation of the average MW of triacylglycerols, phosphatidylethanolamines or phosphatidylcholines and subsequently for calculation of the total amount of lipids in the three classes. The amount of glycerol-3-*P*, phosphoethanolamine and phosphocholine needed for the synthesis of triacylglycerols, phosphatidylethanolamines and phosphatidylcholines was estimated based on the amount the lipid classes in the cells, assuming consumption of 1 mol of glycerol-3-*P*, phosphoethanolamine or phosphocholine per 1 mol of triacylglycerol, phosphatidylethanolamine or phosphatidylcholine, respectively.

Total carbohydrates were measured as described as above. The amount of chitin (g/(g CDW)) in the cells was estimated to be the same as the measured chitin content of *A. oryzae* [8], but corrected for the different ash content of the biomasses of the two species. In the biomass equation of the * T. reesei* model, the amount of chitin is represented as the amount of the monomeric unit, *N*-acetyl-d-glucosamine. The remaining carbohydrates, other than chitin, were presented as glucose units in the model. Furthermore, a trace amount (1e-06 mmol/(g CDW)) of C00096 GDP-mannose and C01083 $\alpha ,\alpha$-trehalose were added for consistency.

The amount of DNA (0.91% (w/w)) was measured as described above. The amount of deoxyribonucleotides in DNA was calculated based on the published GC content of the genome, 52.0% [56].

The amount of RNA in the cells (6.12% (w/w)) was measured as described above. The amount of ribonucleotides in the RNA was estimated based on the nucleotide ratio in 18S-28S pre-rRNA region in the genome (Ensembl, supercontig:GCA_000167675.2:GL985064:1035205:1040758:-1, reverse strand).

The coefficients for C00001 Water, C00002 Adenosine triphosphate, C00008 Adenosine diphosphate, C00009 Organic phosphorous, C00059 sulfate and C00255 riboflavin copied directly from the *S. cerevisiae* model iMM904 [42]. The coefficients were taken as is without any scaling.

Finally, trace amount (1e-06 mmol/(g CDW)) of typical cofactors were added to the biomass equation to ensure the model’s capability to produce these compounds: C00003 Nicotinamide adenine dinucleotide (NAD), C00010 Coenzyme A, C00016 Flavin adenine dinucleotide (FAD), C00051 Glutathione, C00059 sulfate, C00101 tetrahydrofolate, and C00575 3′,5′-cyclic AMP.

### Metabolic model reconstruction

In addition to the reaction database, the CoReCo pipeline required as inputs: (1) genomic data input: genome of the organism of interest and genomes of related species as protein sequences, as well as a phylogenetic tree describing the relationships of the organisms, and (2) reconstruction constraints: source compound list of entities available for the organism to build up its metabolome and biomass through the reactions present and exchange reactions (i.e., metabolites that the organism can take up or secrete).

The basic steps for the reconstruction of the 56 fungi models using CoReCo pipeline were the following:

1. Protein sequences and information about the 56 fungi species were downloaded from JGI.
2. The preprocessing step of the pipeline included adding the protein sequences from the whole-genome models to the file downloaded from Uniprot creating an “augmented SwissProt” as described in "Reference sequence data" section.
3. Two-way BLAST was performed against the “augmented SwissProt” for each of the organisms involved in the reconstruction. The BLAST runs were carried out in parallel under our SGE VTT cluster.
4. GTG was run in parallel under VTT cluster for each of the organisms involved in the model reconstruction.
5. INTERPRO results were downloaded from JGI and reformatted adding the latest GO and E.C. ids. For 49 fungi the INTERPRO results were already computed in [21].
6. A phylogenetic tree was built with CVTree using the protein sequences of all the organisms involved in the model reconstruction.
7. A probabilistic model was built using the results of BLAST, GTG and the phylogenetic tree. Conditional probability distributions were estimated using the E.C. numbers identified with InterProScan in each species as a reference. A score was given to each E.C. for each organism involved in the model reconstruction.
8. Scores were assigned to reactions by copying the score of the E.C. assigned to that reaction. If several E.C.s were annotated to the same reaction, the reaction was assigned the highest score among the scores for the individual enzymes.
9. During the model reconstruction step, the high scoring reactions were added to each model, sequentially filling the gaps from the source compound list. An atom graph was used to find the pathways from the sources to the added high score reaction. This subgraph contains preferably only high score reactions but low score reactions can be added when needed. This step uses two input parameters: $\alpha$ (acceptance threshold), a reaction whose cost is below to this threshold will be added to the subgraph and $\beta$ (rejection threshold), or maximum cost of the complete subgraph. Three different $\alpha$ parameters were tested for this work: 0.3, 0.4 and 0.5. The last one was selected based on the production of the biomass components by the *T. reesei* model.
10. A SBML model was created including all the reactions chosen during the reconstruction step. In addition, two biomass reactions and exchange reactions were added in a post-processing step.
11. Models were tested to identify problems like unrealistic production of compounds and reaction bounds were curated manually. The pipeline was run again from step 5 until no more problems could be identified.

## Additional file

**Additional file 1.** Figure showing a carbon normalized version of the production of each individual biomass compound using FBA.

## Abbreviations

FBA: flux balance analysis
GTG: global trace graph
SBML: systems biology markup language
E.C. number: enzyme commission number
GO: gene ontology
CDW: cell dry weight
JGI: Joint Genome Institute
SGE: sun grid engine
HPLC-ELSD: high-pressure liquid chromatography-evaporative light scattering detector
GC-MS: gas chromatography-mass spectrometry
