# Supplementary figures and images for: Whole-genome metabolic model of Trichoderma reesei built by comparative reconstruction
Source: Biotechnol Biofuels. 2016 Nov 21;9:252. doi: 10.1186/s13068-016-0665-0 (PMC5117618; doi:10.1186/s13068-016-0665-0)

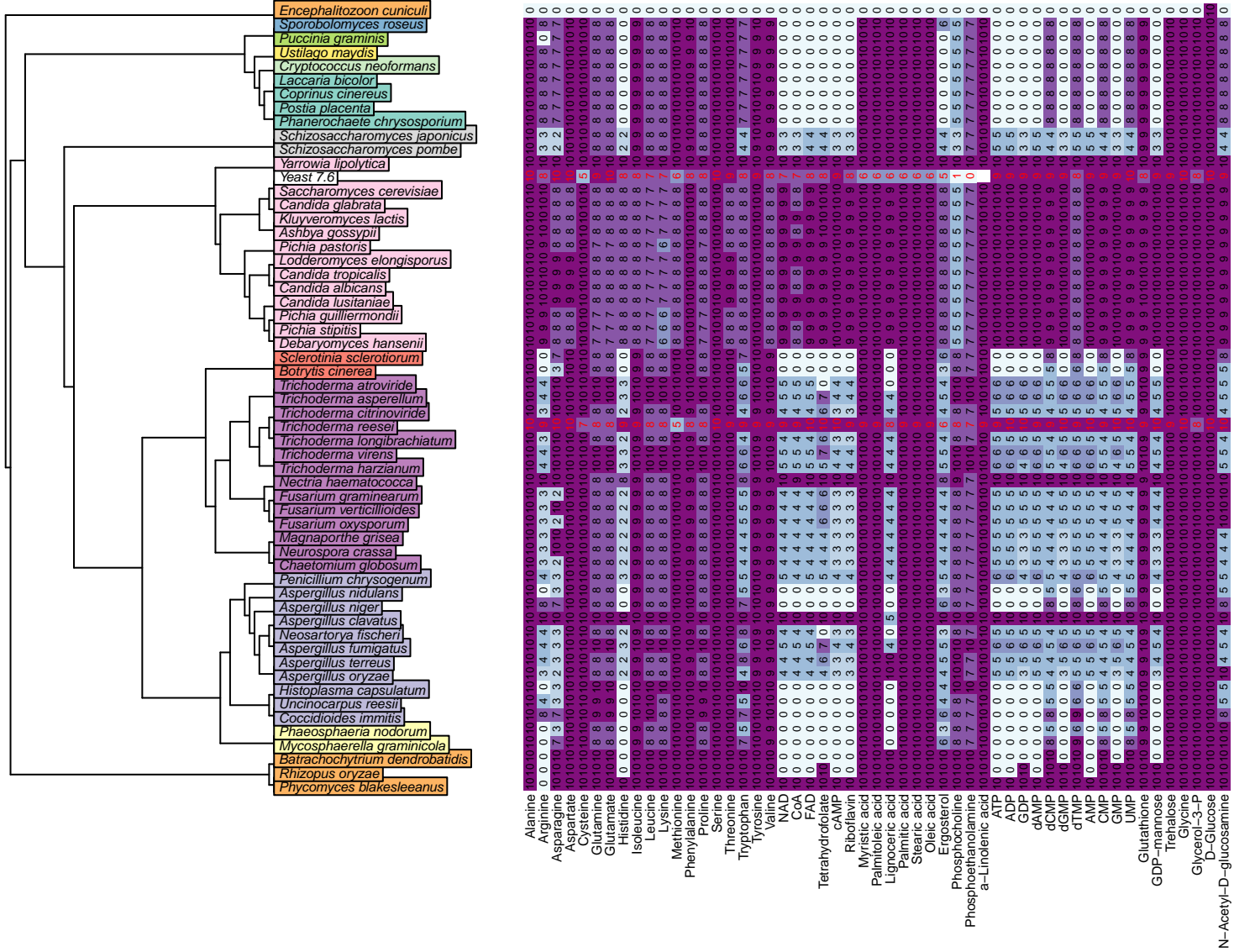

Supplement: Supplementary file 1 — Additional file 1. Figure showing a carbon normalized version of the production of each individual biomass compound using FBA. [file 13068_2016_665_MOESM1_ESM.pdf]
